# Mass Spectrometric Identification of a Novel Factor XIIIa Cross-Linking Site in Fibrinogen

**DOI:** 10.3390/proteomes9040043

**Published:** 2021-11-02

**Authors:** Mariya E. Semkova, J. Justin Hsuan

**Affiliations:** Institute for Liver and Digestive Health, Division of Medicine, University College London, London NW3 2PF, UK; j.hsuan@ucl.ac.uk

**Keywords:** transglutaminases, factor XIIIa, transglutaminase cross-linking, mass spectrometry, fibrinogen

## Abstract

Transglutaminases are a class of enzymes that catalyze the formation of a protein:protein cross-link between a lysine and a glutamine residue. These cross-links play important roles in diverse biological processes. Analysis of cross-linking sites in target proteins is required to elucidate their molecular action on target protein function and the molecular specificity of different transglutaminase isozymes. Mass-spectrometry using settings designed for linear peptide analysis and software designed for the analysis of disulfide bridges and chemical cross-links have previously been employed to identify transglutaminase cross-linking sites in proteins. As no control peptide with which to assess and improve the mass spectrometric analysis of TG cross-linked proteins was available, we developed a method for the enzymatic synthesis of a well-defined transglutaminase cross-linked peptide pair that mimics a predicted tryptic digestion product of collagen I. We then used this model peptide to determine optimal score thresholds for correct peptide identification from y- and b-ion series of fragments produced by collision-induced dissociation. We employed these settings in an analysis of fibrinogen cross-linked by the transglutaminase Factor XIIIa. This approach resulted in identification of a novel cross-linked peptide in the gamma subunit. We discuss the difference in behavior of ions derived from different cross-linked peptide sequences and the consequent demand for a more tailored mass spectrometry approach for cross-linked peptide identification compared to that routinely used for linear peptide analysis.

## 1. Introduction

The transglutaminase (TG) enzymes carry out numerous catalytic and non-catalytic functions [1]. TG isozymes are involved in several vital biological processes including blood coagulation [2], skin-barrier formation [3], cell-envelope formation [4] and extracellular matrix (ECM) assembly [5]. TGs catalyze the condensation of a glutamine and a lysine residue to form a Nε(γ-glutamyl)lysine bond [1]. As a consequence, formation of a protein polymer (de novo direct polymerization) [6], stabilization of non-covalent assemblies by introducing covalent bonds between adjacent subunits—known as enzymatic “spot welding”—or conformational restriction of a folded polypeptide takes place [1,7,8,9]. Apart from their cross-linking activity, this class of enzymes has been reported to carry out other enzymatic activities including protein phosphorylation [10], glutamine deamidation [11], amine incorporation [12] and esterification [13]. Abnormal TG cross-linking activity is implicated in different diseases, including fibrosis [14,15,16], pancreatic cancer [17,18] and celiac disease [19]. For instance, increased cross-linking mediated by transglutaminase 2 (TG2) was found in the ECM during the early inflammatory stage of liver fibrosis [14]. This modification increased ECM abundance by rendering it resistant to remodeling by matrix metalloproteinases [20].

While understanding the substrate specificity of these enzymes is crucial to understanding their mechanism of action, little is known about the cross-linking site preference of TGs. Previous studies have indicated the existence of a canonical sequence around the glutamine residue [21,22,23]. For example, use of a peptide microarray assay demonstrated a strong positive influence of proximal hydrophobic and basic amino acids on the cross-linking reaction rate using a recombinant microbial transglutaminase derived from the *Streptomyces mobaraensis* isozyme [23]. In contrast, TGs seem to have a broad tolerance of the types of amino acid residues surrounding the lysine residue [24,25]. A second criterion that affects the rate of cross-linking is the accessibility of the lysine residue [25]. A slight preference for lysines and glutamines situated on flexible, intrinsically disordered regions of the substrate polypeptide provides further evidence for the importance of steric factors in enzyme specificity [26]. From the studies described above and the fact that different TG isozymes show different substrate specificities [27,28], it is not sufficient to scan potential target protein sequences and structures to identify sites of cross-linking induced by a specific TG. Thus, for each enzyme and substrate there is a need for empirical evidence to identify cross-linking sites.

Several attempts to analyze the site of a TG cross-link have been reported, including the use of Edman degradation [29] and, more recently, mass spectrometry (MS) [30,31,32]. In the latter approach, a purified, cross-linked protein was subjected to tryptic digestion, resulting in a mixture of non-cross-linked (linear) and cross-linked peptides. After peptide separation using liquid chromatography (LC) the sequence of a cross-linked peptide was identified by analyzing its fragmentation spectrum in a tandem MS (MS/MS) experiment. Fragmentation of a cross-linked peptide typically generates a series of cross-linked and linear fragments that result from peptide bond cleavage [33]. Analysis of the MS2 spectrum can then identify the exact position of the cross-linking site within the protein sequence. Similar techniques have been used in studies of protein structure that employ chemical cross-linking followed by digestion and LC-MS/MS analysis [34].

Cross-linked peptides are not identified using established proteomic methods for linear peptide identification using MS/MS and so far, no detailed study of the fragmentation behavior of TG cross-linked tryptic peptides has been reported. We hypothesized that a thorough investigation of their behavior could help develop a method for their identification with high confidence from complex protein digests, ideally using different in silico analyses of the same raw MS/MS data. An important aspect of the identification of unknown cross-linked sequences from complex samples is therefore the identification of MS/MS and software settings required to reliably identify TG cross-linked peptides from large MS datasets [35]. A previous study used MassMatrix software (Columbus, OH, USA) to search for TG cross-linking sites [31]. However, the optimal mass spectrometry and software parameters required for their correct identification using a well-defined cross-linked peptide have not previously been assessed.

In this study, fragmentation analysis using collision induced dissociation (CID) was performed using a synthetic peptide model of a TG cross-link. The first aim was to identify optimal fragmentation conditions based on the resulting number of MS/MS fragments in the y- and b-ion series. The next aim was to find the optimal software parameters for the correct characterization of the peptide model using MassMatrix software. The final aim was to apply these methods to identify the local sequence data and cross-linking position in a fibrinogen γ-γ dimer cross-linked by Factor XIIIa.

## 2. Materials and Methods

All materials were purchased from Sigma (Merk KGaA, Darmstadt, Germany) unless otherwise stated.

### 2.1. Synthesis of a Model TG Cross-Linked Peptide

For the synthesis of the cross-linked peptide, a modified version of the procedure described by Ohtsuka et al. [36] was developed. The 6 mM peptide stock solutions of two peptides with the sequences tert-butyloxycarbonyl (Boc)-EGGKGPR and Boc-SQDGGR (DC Biosciences), respectively, were prepared in an aqueous buffer of 400 mM Tris-HCl, 5 mM CaCl_2_, pH 7.4. The solutions were mixed 1:1 to give a final volume of 50 µL containing 1.5 × 10^−7^ mol of each peptide. A total of 60 mU of guinea pig liver TG2 (product number T5398, 1.18 mU/µL) was added followed by incubation on an orbital mixer at 800 rpm and 37 °C for 3 d. The enzyme activity was terminated by adding 45 µL 0.085% trifluoroacetic acid (TFA, product number 302031). The product was separated from the enzyme via centrifugation through a 3 kDa molecular weight cut-off (MWCO) centrifugal filter (Amicon Ultra, product number UFC5003) for 20 min at 14,000× *g*. The peptide solution (filtrate) was purified using a stage tip (binding capacity: 50 µg). The Boc protecting groups were cleaved in 1:1 TFA:dichloromethane (DCM, product number 270997) on an orbital mixer at 1000 rpm for 2 h at room temperature, followed by solvent evaporation in a vacuum centrifuge. The residue was dissolved in 100 µL 0.1% (*v/v*) formic acid (Darmstadt, Germany, product number 5330020050). The sample composition was analyzed using an LTQ Orbitrap Velos MS system (Thermo Fisher, Waltham, MA, USA) via direct injection (flow rate: 5 µL/min, *m/z* range: 150–2000 Da).

### 2.2. MS Fragmentation Analysis of the Model TG Cross-Linked Peptide 

For each LC-MS/MS run, 10 nmol of the reaction mix from the previous section dissolved in 10 µL 0.1% formic acid was used. Samples were loaded using the autoinjector unit of a nanoAcquity^TM^ ultra performance LC system (Waters). Each sample was first loaded onto a pre-column (nanoAcquity 10K 2G V/M trap column, C18, 5 μm, 180 μm × 20 mm) at a flow rate of 10 µL/min. Peptides were then separated using a separating column (Nikkyo Technos Co. Ltd., Tokio, Japan, C18, 5 μm, 100 μm × 150 mm) at a flow rate of 0.4 μL/min. Separations were performed at 20 °C and the pressure range was 900–1000 psi. The composition of solvent A was aqueous 0.1% formic acid and the composition of solvent B was 0.1% formic acid in acetonitrile (ACN, product number 900667). The solvent gradient was 1% B (0–1 min), 1–60% B (1–15 min), 60–90% B (15–15.01 min), 90% B (15.01–20 min), 1% B (20–30 min). MS/MS analysis was performed using an LTQ Orbitrap Velos instrument (Thermo Fisher, Waltham, MA, USA). The MS1 *m/z* scan range was 300–2000 Da. The 20 most abundant precursor ions from each MS scan were isolated for MS2 analysis and fragmented via CID with helium gas using a collision energy setting of 35. Fragmentation analysis occurred in the linear ion trap with a mass accuracy window of ±0.8 Da. 

### 2.3. Software Analysis

The LC-MS/MS results from the experiments employing ramped CID voltages were analyzed using MassMatrix software (Columbus, OH, USA) [37]. A search was performed against the mouse collagen alpha-1(I) amino acid sequence (source: Uniprot, downloaded 23 April 2018, accession number: P11087). The cleavage enzyme was specified as trypsin with a maximum of 2 missed cleavages per peptide. No variable or fixed modifications were added. A decoy search was performed using the reversed protein sequence. The minimum and maximum peptide lengths were set to 6 and 40 amino acids, respectively. The minimum output pp and pp2 values were 5 and the minimum pp_tag_ value was 1.3. The precursor and fragment mass tolerance were set to ±20 ppm and ±0.8 Da, respectively. The search for a TG cross-link was performed by manually adding a new cross-link to the software database, comprising a lysine and a glutamine residue with a loss of a hydroxyl group (a mass shift of −17.03 Da), which results from the TG cross-linking reaction. The search was performed using the Exploratory Search mode and the maximal number of cross-links per peptide was set to 1. After the search was completed, the results were produced with an inbuilt false discovery rate (FDR) of 0%.

### 2.4. Cross-Linking of Fibrinogen with Factor XIIIa

Cross-linking of fibrinogen with Factor XIIIa occurred at reaction conditions similar to the ones described by Sobel et al. [38]. A total of 3.8 µg of human Factor XIIIa (Haematologic Technologies, Essex Junction, VT, USA, product number HCXIIIA-0165) was added to a solution of 4 mg/mL (200 µg) human fibrinogen (product number F4883) in 0.05 M Tris-HCl and 10 mM CaCl_2_ (pH 7.6) to give a final enzyme concentration of 100 µg/mL (5.6 mU/µL). The reaction mixture was incubated at 37 °C for 110 min. The progress of the reaction was monitored by taking 1 µL aliquots every 10 min. Each aliquot was mixed with loading buffer to a final concentration of 62.5 mM Tris-HCl (Roche, Basel, Switzerland, product number 10812846001), 2.5% SDS (product number L3771), 0.002% bromophenol blue (product number B5525), 10% glycerol (product number G5516), 0.7135 M 2-mercaptoethanol (product number M6250) and incubated at 95 °C for 5 min. Protein separation was performed using SDS-PAGE [39] using a 4–15% TGX^TM^ Precast Protein Gel (Bio-Rad, Hercules, CA, USA, product number 5671085) run on a Criterion electrophoresis chamber (Bio-Rad, Hercules, CA, USA) at 200 V. The gel was fixed in 40% ethanol containing 10% acetic acid for 15 min and rinsed with distilled water for 5 min. The gel was shaken overnight in colloidal Coomassie (QC colloidal Coomassie Stain, Bio-Rad, Hercules, CA, USA, product number 161-0803) at room temperature. Destaining was achieved via incubation with distilled water until a clear background was observed. 

### 2.5. Identification of a Transglutaminase Cross-Linking Site in a Fibrinogen Dimer

Selected gel bands were excised, cut into 1 mm^3^ pieces and each band was transferred to a 1.5-mL tube. Gel pieces were destained by shaking in 200 µL 25 mM ammonium bicarbonate (product number A6141), 50% ACN for 30 min at 37 °C and 1000 rpm. The samples were briefly centrifuged and the solution was removed, followed by repeating the destaining procedure for 5 min. The gel was rehydrated using three washes in 100 µL 25 mM ammonium bicarbonate (pH 8) for 5 min at 25 °C and 1000 rpm. Samples were then treated with Peptide:N-glycosidase F (PNGase F, New England Biolabs, Ipswich, MA, USA, product number P0704S, 500 U per sample) in 100 µL 25 mM ammonium bicarbonate at 37 °C, 1000 rpm for 16 h. After brief centrifugation the samples were incubated with 10 mM DTT (product number D0632) for 2 h at 37 °C and 1000 rpm, followed by 25 mM iodoacetamide (IAA, product number I1149) for 30 min at room temperature in the dark. The solution was removed from the gel, followed by washing in 400 µL 25 mM ammonium bicarbonate and 400 µL ACN (10 min, 1000 rpm, 25 °C). This procedure was repeated once. The samples were dried in a vacuum centrifuge (45 °C, 15 min), rehydrated in 100 µL 25 mM ammonium bicarbonate, and digested with 0.08 µg Endoproteinase Lys-C (Wako, Osaka, Japan, product number 125-05061) for 2 h at 37 °C and 1000 rpm followed by 0.08 µg trypsin (Promega, Madison, WI, product number V5111) overnight at 37 °C and 1000 rpm. After brief centrifugation, the solution was pipetted to a new tube. Extraction of residual peptides from the gel was performed twice, each using 100 µL 5% formic acid (25 °C, 1000 rpm, 15 min). Corresponding extracts were pooled and the samples were evaporated using a vacuum centrifuge at room temperature. Each sample was diluted with 200 µL 0.1% formic acid and purified using a stage tip. The eluate was dissolved in 60 μL 0.1% formic acid, centrifuged (3 min, 16,000× *g*) and 5 µL (365 ng) were used for MS measurements. LC-MS/MS analysis was performed using the same conditions as above with the following gradient: 1% B (0–2 min), 1–6% B (2–6 min), 6–31% B (6–79 min), 31–60% B (79–92 min), 60–90% B (92–93 min), 90% B (93–98 min) and 1% B (98–120 min). Each sample was measured twice using normalized CID fragmentation energy settings of 30 and 35%. Protein identification was performed with the aid of MaxQuant software (version 1.5.5.1, Max Planck Institute of Biochemistry, Planegg, Germany) [40]. MS1 and MS2 spectra were compared with theoretical spectra generated from tryptic in silico digests of human protein sequences with no more than two missed cleavages. The following amino acid modifications that may occur naturally or arise during sample preparation were included in the search: carbamidomethylation (C) (fixed modification); Gln → pyro-Gly (N-terminal), oxidation (M) and acetylation (N-terminal) (variable modifications). Protein quantification was performed by calculating the intensity-based absolute quantification (iBAQ) value. The data were searched against a whole human protein database (source: Uniprot, downloaded 19 July 2016, proteome ID: UP000005640). 

A MassMatrix search for TG cross-linked peptides was performed against the human fibrinogen gamma amino acid sequence (Uniprot, downloaded 12 April 2019, accession number: P02679). The following modifications were added: carbamidomethylation (C) (fixed modification) as well as oxidation (M) and deamidation (Q) (variable modifications). All other parameters were the same as described previously.

## 3. Results

### 3.1. Synthesis of TXLP

**Figure 1:**
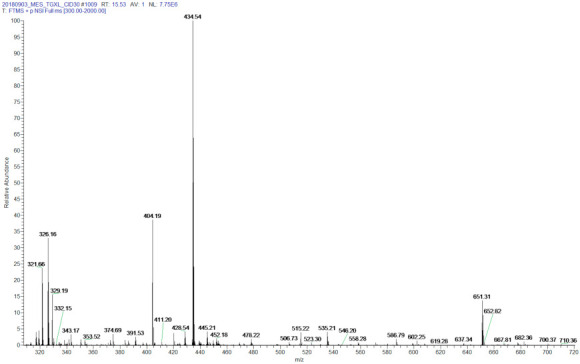
ESI-MS spectrum of the reaction products of TXLP synthesis. Monoisotopic product peaks at 326.16 Da (z = 4), 434.54 Da (z = 3) and 651.31 Da (z = 2) are consistent with the predicted mass of TXLP. No peaks were evident for the precursor peptides (*m/z* = 700.37 and 619.28 at z = 1).

Figure 1 shows the ESI-MS spectrum of the cross-linking reaction products demonstrating the successful synthesis of TXLP. The first aim of this of the study was to synthesize a cross-linked peptide pair with known sequences that would allow (i) an analysis of the collision-induced fragmentation behavior of a model TG cross-linked peptide, and (ii) thresholding of software output scores for different putative sites identified via analysis of fragmentation data. The synthetic peptide pair was selected from a protein sequence that would allow an in silico search against a protein sequence database. The predicted tryptic peptides EGGKGPR (893–899) and SQDGGR (1198–1203) were chosen from the mouse collagen alpha-1(I) sequence. The criteria for peptide selection were (i) the presence of arginine instead of lysine at the C-terminus to avoid side reactions during cross-linking; (ii) linear peptide sequence lengths of no more than 10 amino acids that would ensure efficient HPLC separation using a standard gradient used for LC-MS analysis; and (iii) sufficient hydrophilicity to enable solubilization in water during the cross-linking reaction. Synthesis of the TG cross-linked peptide pair (TXLP, predicted monoisotopic mass 1300.61 Da) was achieved by incubation of the two synthetic peptides with TG2 to catalyze the condensation of the lysine ε-amino group in the EGGKGPR peptide with the glutamine amide group in the SQDGGR peptide. 

### 3.2. Fragmentation Analysis of TXLP

**Figure 2:**
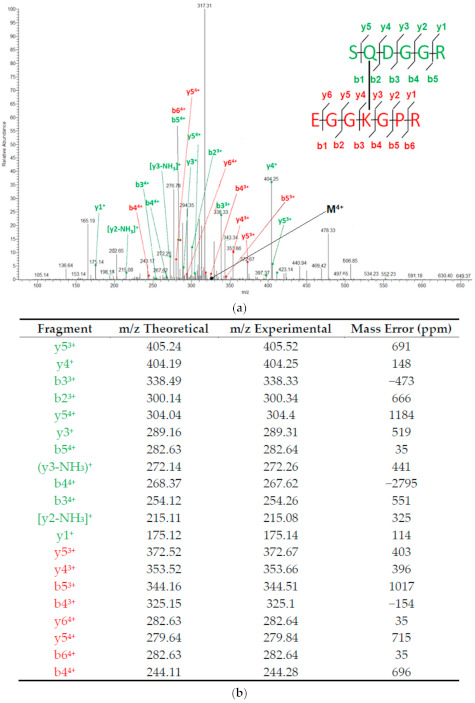
CID fragmentation of the +4-charge state of TXLP. Spectra were collected using CID 35. (**a**), CID fragmentation spectrum (M, precursor peak); (**b**), theoretical and experimental *m/z* values of all identified fragments. The standard mass error of the linear ion trap was 0.8 Da.

To confirm the structure of TXLP and explore the fragmentation pattern of this control TG cross-linked peptide, we generated MS2 spectra using a CID normalized energy setting of 35% (CID 35). This collision energy was selected because it is commonly used for fragmentation of linear tryptic peptides with an LTQ-Orbitrap Velos [41,42,43]. We reasoned that exposure of TXLP to this fragmentation setting would allow us to make a preliminary comparison between the fragmentation behavior of linear and TG cross-linked peptides. We found that the chosen fragmentation conditions generated the majority of ions expected to form from fragmentation of the peptide backbone (Figure 2). As the *m/z* value and intensity of these fragment ions would be subsequently used for the identification of cross-linked sequences, we focused on finding the fragmentation conditions that maximized their abundance. However, increasing or decreasing the collision energy both decreased the intensity of fragment ions (data not shown). We therefore concluded that optimal fragmentation of TXLP occurred at CID 35 and we proceeded to determine whether the fragmentation pattern obtained at CID 35 would allow an in silico identification of the cross-linked site and parent protein.

### 3.3. Identification of TXLP Using MassMatrix

Proteomic software packages widely used for the analysis of linear peptides in trypsin digests, such as MaxQuant, Mascot and Proteome Discoverer, were unable to identify cross-linked peptides. In addition, much of the available bespoke software for cross-linked peptide identification had been designed only for chemical cross-linking and did not include the Lys-Glu isopeptide cross-link. A suitable program called MassMatrix [37] was identified that allowed us to define the properties of the TG cross-link. MS/MS data for the TXLP model peptide, comprising two mouse collagen alpha-1(I) tryptic peptides plus the full-length collagen alpha-1(I) amino acid sequence, were then used to threshold the multiple output scores produced by MassMatrix to allow identification of the cross-linked peptides while minimizing the number of false-positive identifications. 

**Figure 3:**
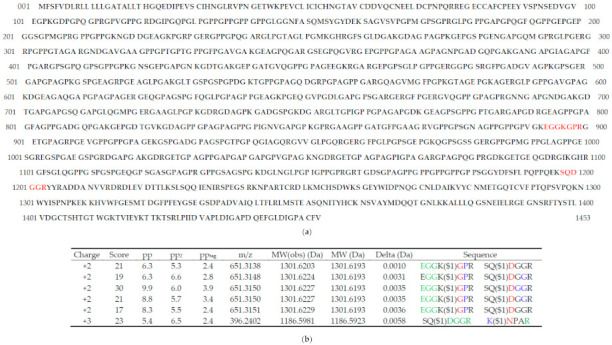

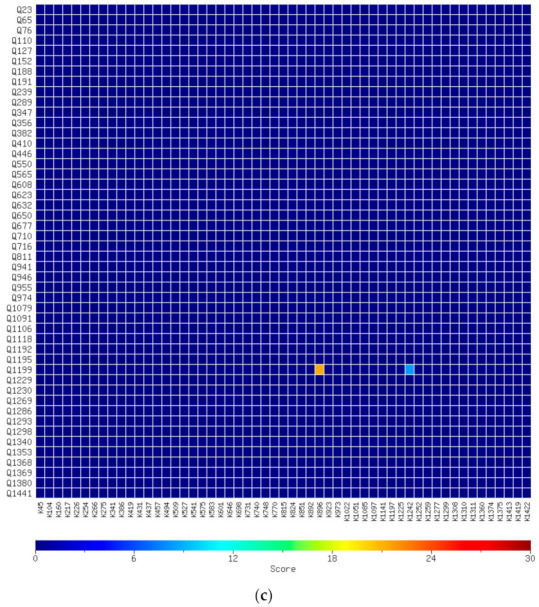
MassMatrix analysis of TXLP LC-MS/MS data. (**a**), The collagen alpha-1(I) amino acid sequence showing the position of the cross-linked peptides (red text). (**b**), Results of each MS2 scan obtained using CID 35 in which cross-linked peptides were identified. The color coding of each amino acid indicates the MS2 fragmentation series in which it was identified (green text, y-series only; blue text, b-series only; red text, y- and b-series; bold type, not identified). The position of the cross-link is indicated ($1) after the cross-linked amino acid. Precursor *m/z* value (*m/z*), theoretical (MW) and observed (MW(obs)) molecular weights, as well as the corresponding mass difference, are shown. (**c**), 2D heatmap of the overall score obtained for each pair of glutamine (Q) and lysine (K) residues in the entire sequence. The strongly identified cross-link position (Q1199-K896) possesses an overall score of approximately 22 while the weakly identified position has a score of approximately 10.

Figure 3 shows a positive identification produced by MassMatrix analysis of the TXLP fragmentation data generated using CID 35. The output from MassMatrix consists of four different scores—three statistical values (pp, pp2 and pp_tag_) and one descriptive value (score) for each spectrum [37]. The value of the score (Figure 3b) is derived from the ratio of the abundance of all matched product ions to the total abundance of all ions in the spectrum and is independent of peptide length. The pp value is the most reliable measure of the degree to which each spectrum fits the indicated peptide sequences, as it considers the number of predicted product ions that match the peaks in that spectrum. Consequently, the pp value implicitly depends on the mass accuracy. The pp2 value is calculated from the total abundance of the matched fragments, while the pp_tag_ value evaluates the match based on selected tags within the peptides (colored in Figure 3b). Hence, the latter two values provide complementary, additional information to the pp value. The overall score displayed in the heat map (Figure 3c) is derived from the values of all statistical parameters of a peptide identification (pp, pp2 and pp_tag_) as well as the number of spectral matches. In addition, the positions of the cross-linked peptides in the collagen alpha-1(I) amino acid sequence are depicted in Figure 3a. 

The MassMatrix result revealed that both cross-linked peptides were correctly identified within the collagen alpha-1(I) protein sequence, and one false-positive identification was also reported. This false-positive could be distinguished from the true-positive identification by the higher number of corresponding MS2 spectra (1 and 5, respectively), the higher pp value (5.4 and 6.3–9.9, respectively), and the higher overall score (10 and 22, respectively). However, we found that a modest change in collision energy from CID 35 to CID 30 (data not shown) produced a false-positive identification with a spectrum pp value (11.4) higher than the true-positive identification spectra (5.9–6.8). The number of spectra at CID 30 (3 true positive vs. 1 false positive) and overall scores (11 true-positive vs. 0 false-positive) qualitatively maintained the differences present at CID 35. Consequently, we inferred that pp, pp2 and pp_tag_ values could not be used to discriminate between true and false-positive identifications as neither consistently exhibited a clear margin between true—and false—positive identifications. Instead, we employed the overall score value to identify true positives, consistent with other types of cross-links. This result is consistent with previous studies using disulfide and bis(sulfosuccinimidyl)suberate)-linked peptides in which putative cross-linked peptide ions with overall scores that appear at or below light blue or cyan on the heatmap (overall score <15 in our CID 35 analysis) were defined as “background” identifications that are considered to be unreliable [37]. In addition, as all false-positive identifications that we obtained at a range of different energy settings were in each case produced by a single MS2 spectrum, we applied an MS2 spectral count threshold of 2 for positive identification in subsequent experiments as it was unclear whether this was included in the overall score threshold.

### 3.4. Identification of TG Cross-Linked Sites in a Fibrinogen γ-γ Dimer

**Figure 4:**
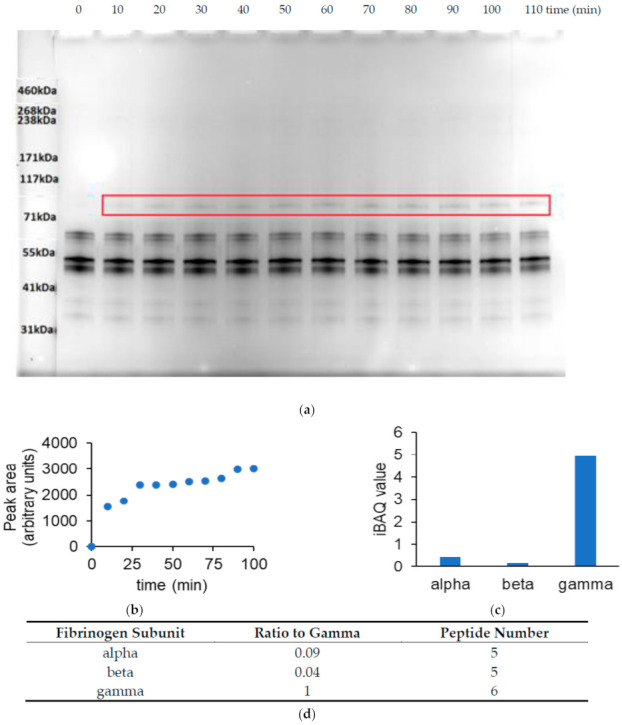
Fibrinogen cross-linking by Factor XIIIa. (**a**), SDS-PAGE analysis of the reaction time course showing the appearance of a specific reaction product (red outline) after 10 min. (**b**), Densitometric analysis of the change in dimer intensity over the reaction period. (**c**), Bar chart of the MaxQuant iBAQ quantification of the abundance of each fibrinogen subunit in the putative dimer band. Samples from the 10, 20 and 30-min reaction times were pooled prior to MS analysis. (**d**), Table providing the iBAQ ratios for each subunit relative to the γ subunit, and the number of peptides identified for each subunit.

To apply the collision energy and software thresholds established using TXLP, the identities of cross-linked sites were explored in a protein that had been enzymatically cross-linked by TG activity. The fibrinogen α_2_β_2_γ_2_ complex is a known target of Factor XIIIa, a TG isozyme that plays a key role in blood clot formation [2]. Figure 4a,b illustrates a time course of human fibrinogen cross-linking by Factor XIIIa, showing the appearance of a high molecular weight band with an apparent size that corresponded to the molecular weight of a γ-γ subunit dimer (approximately 94 kDa). The composition of the dimer was confirmed using in-gel digestion followed by MS analysis (Figure 4c,d). This result was consistent with a previous report that Factor XIIIa preferentially cross-links the γ subunits of purified fibrinogen [2].

To test the ability of MassMatrix to identify the cross-linked sites in the fibrinogen γ-γ dimer, the same MS and software settings previously defined to identify TXLP were used to analyze LC-MS/MS data obtained from the tryptic digest of the gel-purified γ-γ dimer. However, no cross-link position could be identified in a MassMatrix search of the human fibrinogen γ sequence using LC-MS/MS data generated at CID 35. We hypothesized that this might be caused by the absence or insufficiency of expected fragments from the b- and y-ion series in the MS2 spectrum of the cross-linked peptide due to over or under fragmentation. Thus, we tried to identify the peptide by changing the collision energy. This hypothesis was supported by previous literature showing that the optimal collision energy can be affected by the charge state or *m/z* value of a peptide [44,45].

**Figure 5:**
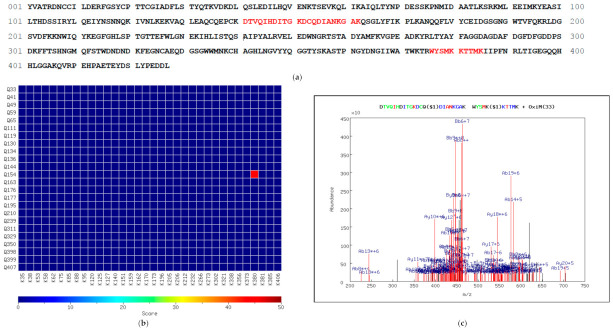

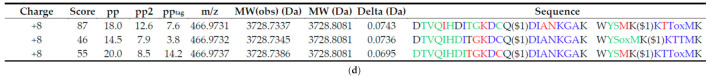
MassMatrix analysis of Factor XIIIa cross-linking sites in fibrinogen. (**a**), Human fibrinogen gamma sequence and (**b**), heatmap showing the position of the two cross-linked peptides identified by MassMatrix. (**c**), MS2 fragmentation spectrum of the cross-linked peptide precursor ion. (**d**), Results for each MS2 scan in which the cross-linked peptide was identified showing the position of the cross-link ($1) after the cross-linked amino acid) and oxidized methionine residues (oxM).

Reducing the collision energy to CID 30 enabled the MassMatrix identification of a single pair of cross-linked peptides, which contained residue Q154 cross-linked to residue K380 (Figure 5). This peptide pair satisfied the proposed threshold criteria for the overall score and the MS2 spectral count (Figure 5b,d). In the three MS2 fragmentation spectra, all of the major peaks were assigned to a predicted fragment from the Q154-K380 cross-linked tryptic peptide, although the low number of abundant peaks and the absence of the precursor ion indicated poor stability and over fragmentation. Both residues are exposed but not intramolecularly juxtaposed in the 3D structure of fibrinogen [46] and therefore likely to be accessible to the cross-linking enzyme and able to mediate dimer formation. A transglutaminase cross-link at K380 has been previously found in ex vivo generated fibrin clots [32]. Moreover, a cross-link site proximal to Q154 (K161) was also reported in this study, suggesting that the corresponding protein region is a likely target for FXIIIa. 

**Figure A1:**
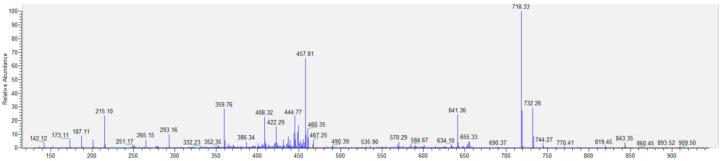
MS/MS fragmentation spectrum of the putative fibrinogen cross-linked peptide obtained using CID 35 (precursor *m/z*: 466.97).

Finally, we returned to the CID 35 data to determine whether we could identify any of the fragment ions based on those identified at CID 30 from the same precursor ion. However, a completely different spectrum was produced from this ion at CID 35 (Figure A1) and we were unable to deduce the structure of any of the fragment ions produced at this collision energy. The complete absence of precursor ions in the spectrum suggested that over fragmentation is even greater than at CID 30.

## 4. Discussion

Our aim is to develop a general workflow for the MS-based identification of TG cross-linking sites in tryptic digests. TG cross-linked peptides produced from proteins digested using chymotrypsin have previously been successfully identified using StavroX software to analyze higher-energy collisional dissociation (HCD) fragmentation by a Q-Exactive mass spectrometer [30]. However, the implications from these data for the identification of tryptic peptides using MassMatrix are limited as tryptic and chymotryptic peptides differ in their fragmentation behavior, and MassMatrix and StavroX employ different analytical methods. TG cross-linked peptides have also previously been identified in a tryptic digest of TG2 cross-linked lacritin using the MassMatrix software [31]; however, the presence of multiple unassigned peaks with relatively high intensities in the fragmentation spectra reduced confidence, and the threshold and sample scores used for positive identification were not reported. An MS-based approach for the identification of transglutaminase cross-linking sites in fibrin clots has been developed by Schmitt et al. [32]. In this study, the characteristic C-terminal γ-γ cross-link site previously discovered by Chen and Doolittle [47] has been successfully identified using MS/MS analysis. Although this result demonstrates the ability of the method to correctly identify transglutaminase cross-linked peptides, the optimal MS conditions and software identification parameters have not been discussed. A study conducted by Schopfer et al. [48] demonstrates how a naturally occurring TG cross-link can be successfully identified by comparing its fragmentation spectrum with that of an identical synthetic peptide by using Protein Prospector [49]. However, this study focuses rather on selecting reliable identifications based on predetermined quality criteria for the MS/MS spectra than finding optimal fragmentation conditions.

To start to address our aim, we synthesized, purified and analyzed TXLP, the first pure, TG cross-linked, control peptide with a defined sequence consistent with a tryptic peptide derived from collagen I. The majority of the TXLP MS2 ions produced at CID 35 (as well as at CID 30 and 40, data not shown) were assigned to fragmentation of the peptide backbone. The majority of the cross-linked fibrinogen peptide MS2 ions produced at CID 30 were similarly assigned, but at CID 35 none of the fragment ions could be identified. These unidentified ions most likely resulted from over fragmentation or rearrangement of the primary fragments and their relative intensity appeared to be very sensitive to collision energy.

Several different properties of a peptide determine which fragment ions are formed by CID [50]. For instance, the presence of basic amino acid residues has a strong influence on fragmentation efficiency and their distribution in a peptide sequence dictates the formation of certain fragments [51]. A TG cross-link modifies a lysine residue by forming a secondary amine at its ε-amino group, which reduces the basicity of lysine at this site. Thus, we expected the fragmentation behavior of cross-linked peptides to be different from the fragmentation behavior of the corresponding linear peptides. These expected differences in fragmentation led us to also expect that the optimal energy required to generate identifiable fragment ions for cross-link identification would differ from that used routinely for linear peptide analysis.

**Figure A2:**
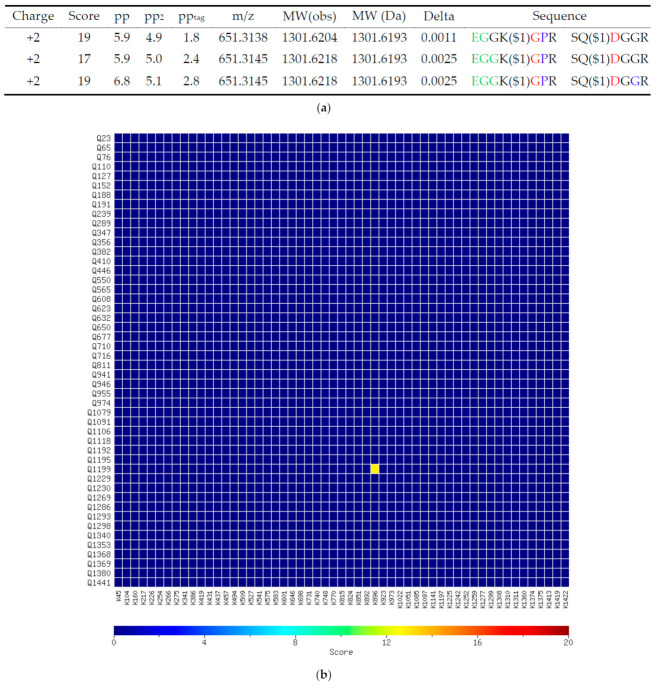
MassMatrix analysis of TXLP LC-MS/MS data obtained using CID 30. (**a**), Results of each MS2 scan in which cross-linked peptides were identified. The color coding of each amino acid indicates the MS2 fragmentation series in which it was identified (green text, y-series only; blue text, b-series only; red text, y- and b-series; bold type, not identified). The position of the cross-link is indicated ($1) after the cross-linked amino acid. Precursor *m/z* value (*m/z*), theoretical (MW) and observed (MW(obs)) molecular weights, as well as the corresponding mass difference, are shown. (**b**), 2D heatmap of the overall score obtained for each pair of glutamine (Q) and lysine (K) residues in the entire mouse collagen alpha-1(I) sequence. The strongly identified cross-link position (Q1199-K896) possesses an overall score of approximately 18.

Based on the results of the CID analysis of TXLP and the fibrinogen cross-linked peptide by MassMatrix, we conclude that different fragmentation energies are needed to obtain the highest possible sequence coverage for different cross-linked peptides. CID 35 gave better results for TXLP in terms of MassMatrix scores and the number of identified MS2 spectra compared with CID 30 (Figure 3 and Figure A2). However, CID 35 was not productive for the fibrinogen cross-linked peptide. This observation may be explained by the charge state difference between these peptides as the +2-charged TXLP was structurally more stable than the +8-charged fibrinogen cross-linked peptide. Thus, lower collision energy (CID 30) was required for optimal fragmentation of the less stable peptide. The effects of peptide charge state, *m/z* value and chemical modification on the energy level necessary for the efficient fragmentation have been previously reported [44,45,52]. We were able to characterize the fragmentation behavior of TXLP MS1 ions with a charge state up to +4. Studies of other peptide sequences are needed to predict the stability and fragmentation patterns of more highly charged ions as a function of CID energy. In general, we expect cross-linked peptides with a minimum charge of +2 to require a normalized CID energy of 35% (similar to typical linear tryptic peptides) and more highly charged cross-linked peptides to require lower CID energies for identification using the Orbitrap Velos mass spectrometer.

New strategies for peptide fragmentation using ramped collision energies have been successfully applied to finding the optimal fragmentation energy for different peptides [45,52,53]. These findings demonstrate how the collision energy level can be optimized for each individual peptide, an advance that has led to the development of the stepped collision energy technique [52]. In this approach, peptides are fragmented at different collision energies followed by combining the fragmentation spectra resulting from each fragmentation energy. This technique allows sequence coverage to be improved for peptides with different optimal fragmentation energies. Alternatively, a data dependent decision tree (DDDT) technique has also been proposed which is based on the discovery that different fragmentation modes are optimal for different precursor charge states and *m/z* values [44,54]. To develop a general method for the analysis of TG cross-linked peptides, a similar approach needs to be explored using a large variety of cross-linked peptides with different charge values in order to determine the optimal collision energy range.

Our assessment of MassMatrix output score thresholds revealed that a high pp value, which depends on a high number of matching fragment ions, was a less reliable parameter than the overall score. As the pp value depends on the total number of theoretical ions, it does not change as the sequence length of the analyte increases. Thus, a comparison between the pp values of TXLP and the cross-linked fibrinogen peptides is appropriate despite the difference in the sequence length. The number of matched product ions is sensitive to mass accuracy and can be affected by noise [55,56]. Thus, the difference in the pp values between the two identified peptides may have arisen, at least in part, from a difference in signal-to-noise ratio. Indeed, when the normalized CID energy was raised from 30 to 35%, an increase in the number of matched ions and an increase in fragment intensities (data not shown) were likely to have contributed to the increase in the maximum pp value obtained for TXLP from 6.8 to 9.9. We found that the most reliable MassMatrix output parameter for true-positive TG cross-link identifications was the overall score (≥blue), with confirmation based on spectral count (>1). This differs from a study of disulfide bridge mapping in which pp_tag_ was reportedly the best output parameter to discriminate true- from false-positive identifications [37].

## 5. Conclusions

In this study, we begin to define a general method for the MS-based mapping of TG cross-linking sites from tryptic digests of complex protein samples. Our hypothesis that a thorough investigation of the fragmentation behavior of TG cross-linked peptides is necessary for their reliable identification was confirmed. CID fragmentation was found to deliver good sequence coverage and MassMatrix analysis of data obtained at CID 30 produced consistent true-positive identifications. As the properties of cross-linked peptides vary widely in the mass spectrometer, future method optimization should include the analysis of diverse model peptides with different sequences and charge states to further our understanding of the effect of these variables on fragmentation behavior. This would allow suitable threshold values to be defined and ensure higher confidence in the identification of novel TG cross-linked peptides.

## Data Availability

The mass spectrometry data have been deposited to the ProteomeXchange Consortium via the PRIDE partner repository with the data set identifier PXD011861.
